# Analysis of All-Cause Hospitalization and Death Among Nonhospitalized Patients With Type 2 Diabetes and SARS-CoV-2 Infection Treated With Molnupiravir or Nirmatrelvir-Ritonavir During the Omicron Wave in Hong Kong

**DOI:** 10.1001/jamanetworkopen.2023.14393

**Published:** 2023-05-19

**Authors:** David T. W. Lui, Matthew S. H. Chung, Eric H. Y. Lau, Kristy T. K. Lau, Ivan C. H. Au, Chi Ho Lee, Yu Cho Woo, Carlos K. H. Wong, Benjamin J. Cowling

**Affiliations:** 1 Department of Medicine, School of Clinical Medicine, Li Ka Shing Faculty of Medicine, The University of Hong Kong, Hong Kong SAR, China; 2 Department of Pharmacology and Pharmacy, Li Ka Shing Faculty of Medicine, The University of Hong Kong, Hong Kong SAR, China; 3 Laboratory of Data Discovery for Health, Hong Kong SAR, China; 4 Department of Family Medicine and Primary Care, School of Clinical Medicine, Li Ka Shing Faculty of Medicine, The University of Hong Kong, Hong Kong SAR, China; 5 World Health Organization Collaborating Centre for Infectious Disease Epidemiology and Control, School of Public Health, Li Ka Shing Faculty of Medicine, The University of Hong Kong, Hong Kong SAR, China

## Abstract

**Importance:**

Diabetes and COVID-19 are both global pandemics, and type 2 diabetes is a common comorbidity in patients with acute COVID-19 and is proven to be a key determinant of COVID-19 prognosis. Molnupiravir and nirmatrelvir-ritonavir are oral antiviral medications recently approved for nonhospitalized patients with mild to moderate COVID-19, following demonstration of their efficacies in reducing adverse outcomes of the disease; it is crucial to clarify whether both oral antiviral medications are efficacious in a population consisting exclusively of patients with type 2 diabetes.

**Objective:**

To evaluate the effectiveness of molnupiravir and nirmatrelvir-ritonavir in a contemporary population-based cohort comprising exclusively nonhospitalized patients with type 2 diabetes and SARS-CoV-2 infection.

**Design, Setting, and Participants:**

This retrospective cohort study was performed using population-based electronic medical record data for patients in Hong Kong with type 2 diabetes and confirmed SARS-CoV-2 infection between February 26 and October 23, 2022. Each patient was followed up until death, outcome event, crossover of oral antiviral treatment, or end of the observational period (October 30, 2022), whichever came first. Outpatient oral antiviral users were divided into molnupiravir and nirmatrelvir-ritonavir treatment groups, respectively, and nontreated control participants were matched through 1:1 propensity score matching. Data analysis was performed on March 22, 2023.

**Exposures:**

Molnupiravir (800 mg twice daily for 5 days) or nirmatrelvir-ritonavir (300 mg nirmatrelvir and 100 mg ritonavir twice daily for 5 days, or 150 mg nirmatrelvir and 100 mg ritonavir for patients with an estimated glomerular filtration rate of 30-59 mL/min per 1.73 m^2^).

**Main Outcomes and Measures:**

The primary outcome was a composite of all-cause mortality and/or hospitalization. The secondary outcome was in-hospital disease progression. Hazard ratios (HRs) were estimated with Cox regression.

**Results:**

This study identified 22 098 patients with type 2 diabetes and COVID-19. A total of 3390 patients received molnupiravir and 2877 received nirmatrelvir-ritonavir in the community setting. After application of exclusion criteria followed by 1:1 propensity score matching, this study comprised 2 groups. One group included 921 molnupiravir users (487 men [52.9%]), with a mean (SD) age of 76.7 (10.8) years, and 921 control participants (482 men [52.3%]), with a mean (SD) age of 76.6 (11.7) years. The other group included 793 nirmatrelvir-ritonavir users (401 men [50.6%]), with a mean (SD) age of 71.7 (11.5) years, and 793 control participants (395 men [49.8%]), with a mean (SD) age of 71.9 (11.6) years. At a median follow-up of 102 days (IQR, 56-225 days), molnupiravir use was associated with a lower risk of all-cause mortality and/or hospitalization (HR, 0.71 [95% CI, 0.64-0.79]; *P* < .001) and in-hospital disease progression (HR, 0.49 [95% CI, 0.35-0.69]; *P* < .001) compared with nonuse. At a median follow-up of 85 days (IQR, 56-216 days), nirmatrelvir-ritonavir use was associated with a lower risk of all-cause mortality and/or hospitalization (HR, 0.71 [95% CI, 0.63-0.80]; *P* < .001) and a nonsignificantly lower risk of in-hospital disease progression (HR, 0.92 [95% CI, 0.59-1.44]; *P* = .73) compared with nonuse.

**Conclusions and Relevance:**

These findings suggest that both molnupiravir and nirmatrelvir-ritonavir oral antiviral medications were associated with a lower risk of all-cause mortality and hospitalization among patients with COVID-19 and type 2 diabetes. Further studies in specific populations, such as individuals in residential care homes and individuals with chronic kidney disease, are suggested.

## Key Points

**Question:**

Are oral antiviral medications such as molnupiravir and nirmatrelvir-ritonavir associated with improved outcomes among patients with type 2 diabetes and COVID-19?

**Findings:**

In this cohort study of 22 098 patients in Hong Kong with type 2 diabetes and confirmed SARS-CoV-2 infection, oral antiviral use was associated with a 29% lower risk of all-cause mortality and/or hospitalization for both molnupiravir users and nirmatrelvir-ritonavir users compared with respective control participants.

**Meaning:**

These findings suggest that treatment with molnupiravir or nirmatrelvir-ritonavir was associated with a lower risk of all-cause mortality and hospitalization among patients with COVID-19 and type 2 diabetes.

## Teaser

This cohort study examines the effectiveness of molnupiravir and nirmatrelvir-ritonavir in nonhospitalized patients with type 2 diabetes and SARS-CoV-2 infection during the Omicron wave in Hong Kong.

## Introduction

The COVID-19 pandemic, caused by SARS-CoV-2 infection, has affected more than 630 million individuals worldwide and caused more than 6.6 million deaths.^1^ Diabetes is another pandemic affecting approximately 463 million individuals globally.^2^ Unfortunately, type 2 diabetes is a common comorbidity in patients with acute COVID-19, and evidence has established type 2 diabetes as a key determinant of COVID-19 prognosis.^2,3^ While individuals with type 2 diabetes have more coexisting comorbidities (eg, obesity and kidney impairment) that compound adverse COVID-19 outcomes, diabetes per se also causes worse COVID-19 outcomes as evidenced by the increased risk of adverse outcomes with worse glycemic control.^4^ The pathophysiologic mechanisms involve reduced viral clearance and an enhanced inflammatory state.^5^ Although COVID-19 vaccination has proven efficacy in protection against severe disease, patients with diabetes may have an impaired immune response to vaccination against COVID-19.^6^ Hence, among patients with type 2 diabetes and SARS-CoV-2 infection, it is vital to have effective antiviral medications to reduce adverse outcomes of COVID-19.

Molnupiravir and nirmatrelvir-ritonavir are 2 oral antiviral medications recently approved for nonhospitalized patients with mild to moderate COVID-19, following the landmark randomized clinical trials demonstrating their efficacies in reducing adverse outcomes of the disease.^7,8^ Molnupiravir and nirmatrelvir-ritonavir are indicated for patients with COVID-19 and risk factors for progression to severe disease, one of which is diabetes.^9^ Nonetheless, patients with diabetes only represented up to 15% of the included participants in the 2 landmark trials.^7,8^ Furthermore, in the trial involving molnupiravir,^7^ the point estimate for the risk difference of hospitalization or death even favored placebo over molnupiravir. Indeed, COVID-19 consists of 2 phases: the early viral replication and the late inflammatory phase.^10^ Patients with type 2 diabetes have several potential pathophysiologic mechanisms for worse COVID-19–related outcomes, such as enhanced viral entry, decreased T-cell function with reduced viral clearance, and hyperinflammation.^11^ In addition, use of antidiabetic agents may affect COVID-19 outcomes and should be considered when evaluating the efficacy of oral antiviral medications in patients with type 2 diabetes and COVID-19.^12^ Hence, it is crucial to clarify whether molnupiravir and nirmatrelvir-ritonavir are efficacious in a population consisting exclusively of patients with type 2 diabetes and whether the efficacy of these medications extends to the entire population with type 2 diabetes. These results will provide valuable data to guide clinicians in prescribing antiviral medications to this at-risk group of patients. To this end, we carried out this population-based analysis to assess the clinical effectiveness of molnupiravir and nirmatrelvir-ritonavir among community-dwelling outpatients with type 2 diabetes and SARS-CoV-2 infection during the Omicron wave in Hong Kong.

## Methods

### Study Design and Data Sources

This retrospective cohort study was approved by the institutional review board of the University of Hong Kong and Hospital Authority Hong Kong West Cluster, which waived the need for individual patient-informed consent owing to the use of anonymized data. The study followed the Strengthening the Reporting of Observational Studies in Epidemiology (STROBE) reporting guideline.

We included patients in Hong Kong with type 2 diabetes and confirmed SARS-CoV-2 infection between February 26 and October 23, 2022, identified from population-based electronic medical records. This was the period in which the COVID-19 pandemic in Hong Kong was dominated by the SARS-CoV-2 Omicron variant.^13^ A positive reverse transcription–polymerase chain reaction or rapid antigen test result confirmed SARS-CoV-2 infection. Electronic medical records of all patients with COVID-19 in the territory were retrieved from the Hospital Authority. The Hospital Authority database has been used extensively for conducting observational cohort studies to evaluate drug therapies for COVID-19 at a population level.^14,15,16,17^ In this retrospective territory-wide cohort of patients with confirmed SARS-CoV-2 infection, we identified individuals with type 2 diabetes by using *International Classification of Primary Care, Second Edition* (*ICPC-2*) code T89 or T90 or *International Classification of Diseases, 9th Revision, Clinical Modification* (*ICD-9-CM*) code 250.xx, with subsequent exclusion of patients with type 1 diabetes (*ICPC-2* code T89 or *ICD-9-CM* code 250.x1 or 250.x3).

### Study Population

Molnupiravir has been locally available for prescription since February 26, 2022, and nirmatrelvir-ritonavir since March 16, 2022. According to Hong Kong Hospital Authority COVID-19 clinical management guidelines,^18^ patients with the following conditions were recommended to receive molnupiravir or nirmatrelvir-ritonavir: (1) mild symptoms; (2) risk factors for progression to severe disease, including diabetes, obesity (body mass index [calculated as weight in kilograms divided by height in meters squared] ≥30), age 60 years or older, immunocompromised state, underlying chronic illnesses, or not fully vaccinated; and (3) early disease stage (within 5 days of symptom onset). Nirmatrelvir-ritonavir was preferred over molnupiravir unless patients were taking concomitant medications contraindicated for nirmatrelvir-ritonavir. Outpatient oral antiviral users were divided into 2 groups: one received molnupiravir (800 mg twice daily for 5 days) and the other received nirmatrelvir-ritonavir (300 mg nirmatrelvir and 100 mg ritonavir twice daily for 5 days, or 150 mg nirmatrelvir and 100 mg ritonavir if their estimated glomerular filtration rate [eGFR] was 30-59 mL/min per 1.73 m^2^)^18^ based on their drug prescription and dispensing records. The index date was defined as the date of confirmed SARS-CoV-2 infection or symptom onset, whichever occurred earlier. The control cohort was selected from patients with confirmed SARS-CoV-2 infection before admission and those who did not receive any oral antiviral medications in the outpatient setting during the observational period. Each patient was followed up from the index date until death, outcome event occurrence, crossover of oral antiviral treatment, or the end of the observational period (October 30, 2022), whichever came first. We excluded patients who (1) were aged younger than 18 years, (2) were admitted to the hospital before diagnosis of SARS-CoV-2 infection or died on or before diagnosis of SARS-CoV-2 infection, (3) were taking medications with contraindications to nirmatrelvir-ritonavir,^19^ (4) resided in residential care homes for older individuals, (5) had severe kidney impairment^20^ (eGFR <30 mL/min per 1.73 m^2^ or dialysis or kidney transplantation), (6) had severe liver impairment^20^ (cirrhosis, hepatocellular carcinoma, or liver transplantation), or (7) initiated oral antiviral medications more than 5 days after symptom onset.

### Covariates

Baseline characteristics, including age, sex, date of SARS-CoV-2 infection (dichotomized as before September 1, 2022, and on or after September 1, 2022 [the date that marked the beginning of the sixth wave of the pandemic in Hong Kong]), COVID-19 vaccination status, and preexisting comorbidities (Charlson Comorbidity Index [CCI] and individual comorbidities), were captured based on *ICD-9-CM* diagnosis codes, *ICPC-2* codes, treatment records, and clinical parameters (eTable 4 in Supplement 1). Baseline medication use was also retrieved. Fully vaccinated patients were defined as those who had received at least 2 doses of BNT162b2 messenger RNA vaccine (Comirnaty; Pfizer-BioNTech) or 3 doses of inactivated Vero cell COVID-19 vaccine (CoronaVac; Sinovac).^21^

### Outcomes

The primary outcome was a composite outcome of all-cause mortality and/or hospitalization. Secondary outcomes included (1) all-cause mortality, (2) all-cause hospitalization, and (3) a composite outcome of in-hospital disease progression (in-hospital mortality, invasive mechanical ventilation, or intensive care unit admission). For all-cause hospitalization, only the first hospital admission after diagnosis of SARS-CoV-2 infection was used if a patient had multiple hospital admissions.

### Statistical Analysis

We used propensity score models conditional on age, sex, CCI score, preexisting comorbidities, medication use, and COVID-19 vaccination status in a logistic regression model. We used 1:1 propensity score matching without replacement using a caliper width of 0.05. Standardized mean differences (SMDs) of each covariate between groups before and after propensity score matching were calculated and were interpreted as balanced when the SMD was less than 0.1.^22^ Cox regression models were used to estimate each outcome between oral antiviral users and their respective matched nonusers, expressed in terms of hazard ratios (HRs) and 95% CIs. The goodness of fit for Cox regression models was tested using Wald tests, and the significant results supported that our Cox regression models were well fit.

Subgroup analyses assessed interactions by age (≤65 vs >65 years), sex, COVID-19 vaccination status (fully vaccinated vs not fully vaccinated), baseline insulin use, presence of chronic kidney disease, and presence of diabetic complications. Sensitivity analyses were performed as follows: (1) including patients given oral antiviral medications within 2 days since the index date, to assess the impact of potential immortal time bias; (2) limiting follow-up to a maximum of 30 days; (3) adjusting the propensity score model to exclude variables of individual comorbidities and medication use; (4) adjusting the propensity score model with a wider caliper width of 0.1 and increased ratio of 1:2; and (5) including molnupiravir users who were taking medications contraindicated to nirmatrelvir-ritonavir.

All statistical analyses were performed using Stata, version 17 (StataCorp LLC). All significance tests were 2 tailed, where *P* < .05 was considered statistically significant. Data analysis was performed on March 22, 2023.

## Results

**Figure 1.**
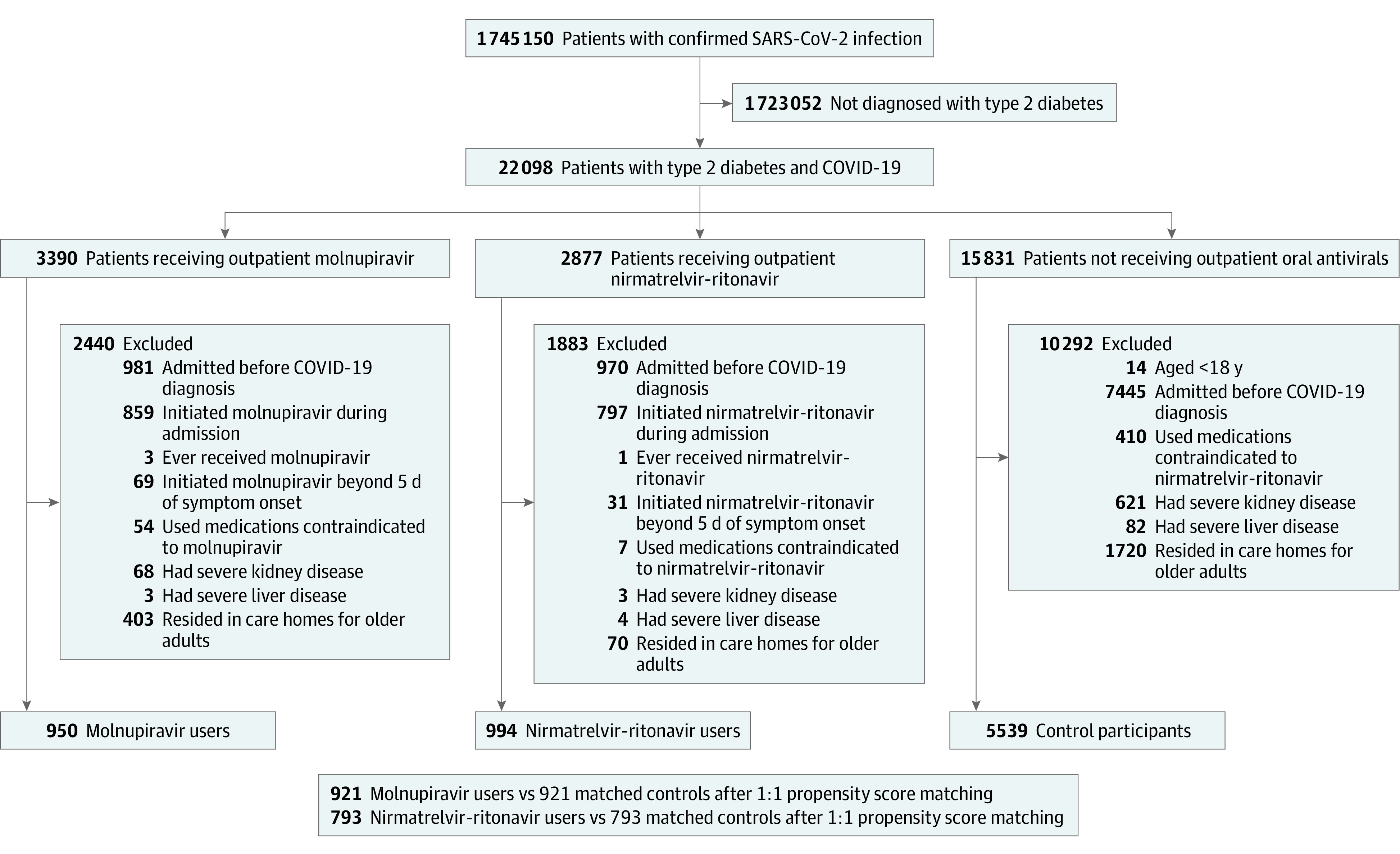
Inclusion and Exclusion Criteria for Patients With Type 2 Diabetes and COVID-19 Who Received Outpatient Molnupiravir or Nirmatrelvir-Ritonavir vs Control Participants

A total of 22 098 patients with type 2 diabetes and confirmed SARS-CoV-2 infection between February 26 and October 23, 2022, in Hong Kong were identified (Figure 1). In this study cohort, 11 924 patients (54.0%) were men and 10 174 (46.0%) were women; their mean (SD) age was 75.9 (12.5) years. A total of 3390 patients received molnupiravir and 2877 received nirmatrelvir-ritonavir in the community setting. After application of exclusion criteria followed by 1:1 propensity score matching, the study comprised 2 groups. One group included 921 molnupiravir users (487 men [52.9%] and 434 women [47.1%]), with a mean (SD) age of 76.7 (10.8) years, and 921 matched control participants (482 men [52.3%] and 439 women [47.7%]), with a mean (SD) age of 76.6 (11.7) years. The other group included 793 nirmatrelvir-ritonavir users (401 men [50.6%] and 392 women [49.4%]), with a mean (SD) age of 71.7 (11.5) years, and 793 matched control participants (395 men [49.8%] and 398 women [50.2%]), with a mean (SD) age of 71.9 (11.6) years. Baseline characteristics of molnupiravir users, nirmatrelvir-ritonavir users, and control participants before propensity score matching are presented in eTable 1 in Supplement 1. Most patients were older than 65 years. A total of 878 molnupiravir users (95.3%) and 781 nirmatrelvir-ritonavir users (98.5%) were prescribed the standard 5-day course of oral antiviral medications. The most common comorbidity was hypertension. Regarding diabetes-specific characteristics, 420 (26.5%) to 689 (37.4%) of all individuals were insulin users and 256 (16.1%) to 541 (29.4%) of all individuals had diabetic complications. In this study cohort, none of the included patients had previous SARS-CoV-2 infection.

Baseline characteristics of oral antiviral users and respective matched control participants were balanced after 1:1 propensity score matching (Table 1), and the distribution of propensity scores between the oral antiviral and respective control groups highly overlapped (eFigure 1 in Supplement 1). Compared with nirmatrelvir-ritonavir users, molnupiravir users were older with more preexisting comorbidities, were less likely to be fully vaccinated or boosted, and were more likely to be insulin users and have more diabetic complications. There was no difference in age and CCI score of patients who received molnupiravir before and after nirmatrelvir-ritonavir approval.

**Table 1.** Baseline Characteristics of Patients With Type 2 Diabetes and COVID-19 in the Molnupiravir, Nirmatrelvir-Ritonavir, and Respective Matched Control Groups After 1:1 Propensity-Score Matching^a^

Characteristic	Molnupiravir			Nirmatrelvir-ritonavir		
	Oral antiviral group (n = 921)	Control group (n = 921)	SMD	Oral antiviral group (n = 793)	Control group (n = 793)	SMD
Age, y						
Mean (SD)	76.7 (10.8)	76.6 (11.7)	0.01	71.7 (11.5)	71.9 (11.6)	0.02
18-40	6 (0.7)	13 (1.4)	0.01	6 (0.8)	6 (0.8)	0.04
41-65	118 (12.8)	108 (11.7)		215 (27.1)	200 (25.2)	
>65	797 (86.5)	800 (86.9)		572 (72.1)	587 (74.0)	
Sex						
Male	487 (52.9)	482 (52.3)	0.01	401 (50.6)	395 (49.8)	0.02
Female	434 (47.1)	439 (47.7)		392 (49.4)	398 (50.2)	
Time of SARS-CoV-2 infection						
Before September 1, 2022	223 (24.2)	229 (24.9)	0.02	226 (28.5)	234 (29.5)	0.02
On or after September 1, 2022	698 (75.8)	692 (75.1)		567 (71.5)	559 (70.5)	
Preexisting comorbidity						
CCI, mean (SD)^b^	5.7 (1.6)	5.7 (1.7)	0.01	5.0 (1.4)	5.0 (1.5)	0.04
0-4	153 (16.6)	155 (16.8)	0.03	278 (35.1)	273 (34.4)	0.04
5-6	537 (58.3)	524 (56.9)		428 (54.0)	423 (53.3)	
7-16	231 (25.1)	242 (26.3)		87 (11.0)	97 (12.2)	
Hypertension	850 (92.3)	846 (91.9)	0.02	668 (84.2)	670 (84.5)	0.01
Chronic lung disease	118 (12.8)	125 (13.6)	0.02	57 (7.2)	64 (8.1)	0.03
Chronic heart disease	238 (25.8)	237 (25.7)	0	116 (14.6)	122 (15.4)	0.02
Chronic kidney disease	55 (6.0)	61 (6.6)	0.03	9 (1.1)	8 (1.0)	0.01
Liver disease	35 (3.8)	38 (4.1)	0.02	35 (4.4)	33 (4.2)	0.01
Malignant neoplasm	46 (5.0)	53 (5.8)	0.03	32 (4.0)	39 (4.9)	0.04
Gout	20 (2.2)	28 (3.0)	0.05	10 (1.3)	9 (1.1)	0.01
Obesity	59 (6.4)	70 (7.6)	0.05	64 (8.1)	54 (6.8)	0.05
Obstructive sleep apnea	0	0	NA	0	0	NA
Depression	1 (0.1)	1 (0.1)	0	0	0	NA
Severe hypoglycemia	71 (7.7)	83 (9.0)	0.05	46 (5.8)	40 (5.0)	0.03
Diabetic complication	270 (29.3)	271 (29.4)	0	126 (15.9)	130 (16.4)	0.01
Macrovascular disease	254 (27.6)	255 (27.7)	0	118 (14.9)	120 (15.1)	0.01
Coronary heart disease	128 (13.9)	129 (14.0)	0	57 (7.2)	54 (6.8)	0.01
Heart failure	65 (7.1)	68 (7.4)	0.01	11 (1.4)	21 (2.6)	0.09
Stroke	111 (12.1)	104 (11.3)	0.02	60 (7.6)	54 (6.8)	0.03
Peripheral vascular disease	6 (0.7)	6 (0.7)	0	4 (0.5)	4 (0.5)	0
Microvascular disease	29 (3.1)	28 (3.0)	0.01	11 (1.4)	14 (1.8)	0.03
Nonproliferative diabetic retinopathy	22 (2.4)	20 (2.2)	0.01	8 (1.0)	9 (1.1)	0.01
Sight threatening diabetic retinopathy	9 (1.0)	8 (0.9)	0.01	3 (0.4)	3 (0.4)	0
Neuropathy	4 (0.4)	4 (0.4)	0	3 (0.4)	2 (0.3)	0.02
End-stage kidney disease	0	0	NA	0	0	NA
COVID-19 vaccination status^c^						
Not fully vaccinated	534 (58.0)	518 (56.2)	0.04	329 (41.5)	332 (41.9)	0.03
Fully vaccinated but not boosted	251 (27.3)	262 (28.5)		289 (36.4)	296 (37.3)	
Boosted	136 (14.8)	141 (15.3)		175 (22.1)	165 (20.8)	
No. of COVID-19 vaccine doses received						
0-2	332 (36.0)	364 (39.5)	0.07	255 (32.2)	261 (32.9)	0.02
≥3	589 (64.0)	557 (60.5)		538 (67.8)	532 (67.1)	
Long-term medication use						
ACEIs or ARBs	572 (62.1)	559 (60.7)	0.03	408 (51.5)	406 (51.2)	0.01
Anticoagulants	249 (27.0)	247 (26.8)	0	90 (11.3)	86 (10.8)	0.02
Antiplatelets	366 (39.7)	344 (37.4)	0.05	215 (27.1)	227 (28.6)	0.03
Lipid-lowering agents	681 (73.9)	679 (73.7)	0	518 (65.3)	513 (64.7)	0.01
NSAIDs	438 (47.6)	419 (45.5)	0.04	301 (38.0)	314 (39.6)	0.03
β-blockers	370 (40.2)	351 (38.1)	0.04	215 (27.1)	212 (26.7)	0.01
Calcium-channel blockers	596 (64.7)	611 (66.3)	0.03	425 (53.6)	453 (57.1)	0.07
Diuretics	279 (30.3)	286 (31.1)	0.02	135 (17.0)	137 (17.3)	0.01
Antidepressants	98 (10.6)	100 (10.9)	0.01	37 (4.7)	40 (5.0)	0.02
Antidiabetic regimen						
Noninsulin users, No. of agents						
1	209 (22.7)	216 (23.5)	0.04	207 (26.1)	193 (24.3)	0.05
2	134 (14.6)	142 (15.4)		131 (16.5)	133 (16.8)	
>2	79 (8.6)	74 (8.0)		77 (9.7)	75 (9.5)	
Insulin	350 (38.0)	339 (36.8)	0.02	209 (26.4)	211 (26.6)	0.01
Antidiabetic medication						
GLP1RA	14 (1.5)	9 (1.0)	0.05	9 (1.1)	5 (0.6)	0.05
Metformin	617 (67.0)	612 (66.4)	0.01	583 (73.5)	576 (72.6)	0.02
Sulfonylurea	410 (44.5)	414 (45.0)	0.01	302 (38.1)	311 (39.2)	0.02
Thiazolidinedione	75 (8.1)	68 (7.4)	0.03	76 (9.6)	79 (10.0)	0.01
Acarbose	5 (0.5)	2 (0.2)	0.05	6 (0.8)	5 (0.6)	0.02
SGLT2i	110 (11.9)	103 (11.2)	0.02	65 (8.2)	56 (7.1)	0.04
DPP4i	256 (27.8)	224 (24.3)	0.08	156 (19.7)	150 (18.9)	0.02

Abbreviations: ACEI, angiotensin-converting enzyme inhibitor; ARB, angiotensin receptor blocker; CCI, Charlson Comorbidity Index; DPP4i, dipeptidyl peptidase 4 inhibitor; GLP1RA, glucagon-like peptide 1 receptor agonist; NA, not applicable; NSAID, nonsteroidal anti-inflammatory drug; SGLT2i, sodium-glucose cotransporter-2 inhibitor; SMD, standardized mean difference.

^a^ Unless indicated otherwise, values are presented as No. (%) of participants.

^b^ The CCI calculation did not include AIDS.

^c^ Fully vaccinated but not boosted patients were defined as those who had received 2 doses of BNT162b2 messenger RNA vaccine (Comirnaty; Pfizer-BioNTech) or 3 doses of inactivated Vero cell COVID-19 vaccine (CoronaVac; Sinovac); boosted patients were defined as those who had received at least 3 doses of BNT162b2 or 4 doses of inactivated Vero cell COVID-19 vaccine.

### Risks of Outcomes Associated With Molnupiravir and Nirmatrelvir-Ritonavir Use

**Figure 2.**
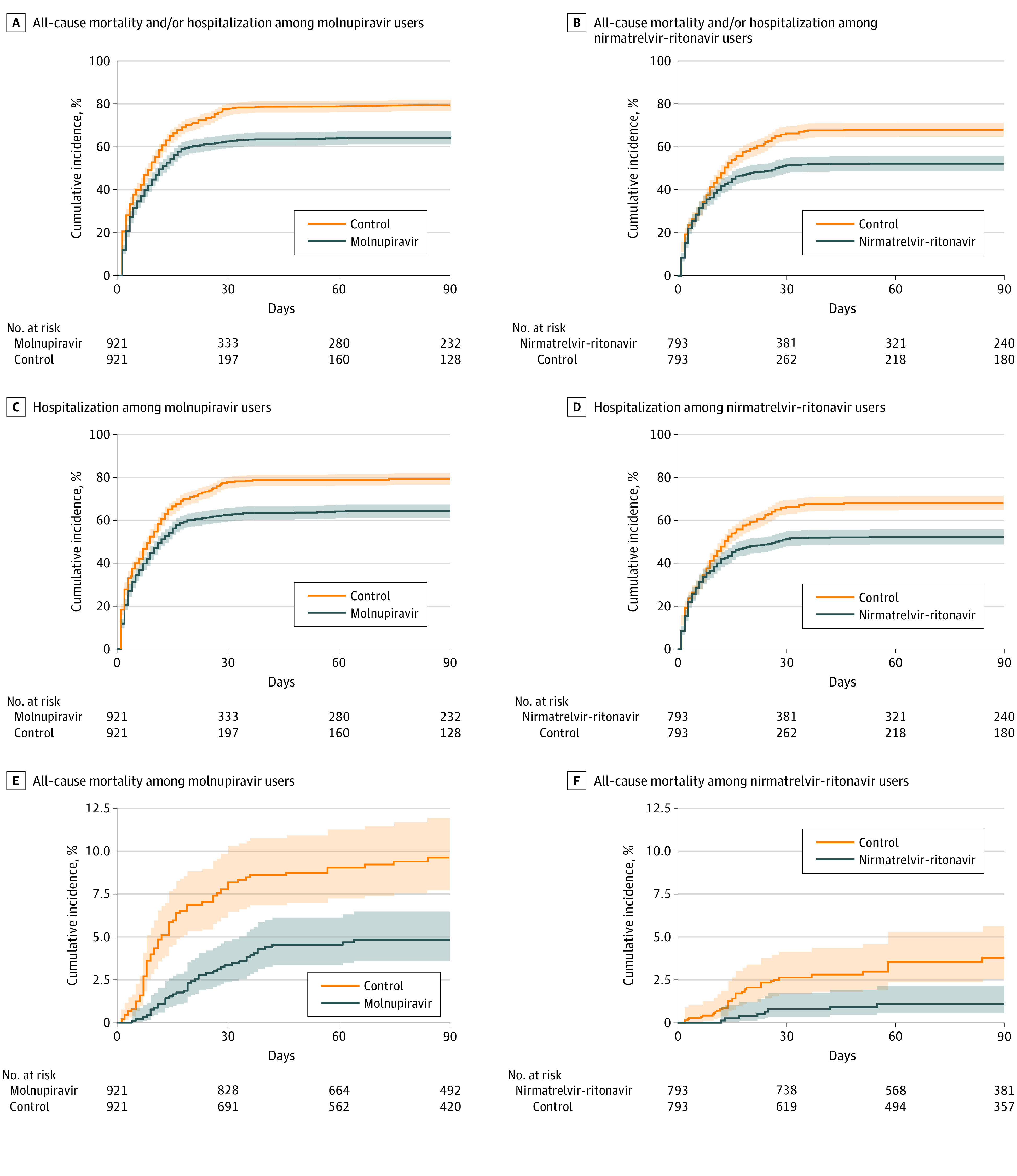
Cumulative Incidence of Primary Outcome, Hospitalization, and All-Cause Mortality for Molnupiravir Users, Nirmatrelvir-Ritonavir Users, and Respective Matched Control Groups A, C, and E, All-cause mortality and/or hospitalization (A), all-cause hospitalization (C), and all-cause mortality (E) among outpatient molnupiravir users vs matched control participants. B, D, and F, All-cause mortality and/or hospitalization (B), all-cause hospitalization (D), and all-cause mortality (F) among outpatient nirmatrelvir-ritonavir users vs matched control participants.

The median follow-up for molnupiravir and nirmatrelvir-ritonavir users was 102 days (IQR, 56-225 days) and 85 days (IQR, 56-216 days), respectively. The cumulative incidence of the composite outcome of all-cause mortality and/or hospitalization, all-cause mortality, hospitalization, and composite in-hospital disease progression between the oral antiviral and respective control groups is shown in Figure 2 and eFigure 2 in Supplement 1.

For the molnupiravir group, crude incidence rates of the composite outcome of all-cause mortality and/or hospitalization were 1024.9 (95% CI, 944.0-1110.8) per 100 000 person-days among molnupiravir users and 1952.4 (95% CI, 1813.4-2099.2) per 100 000 person-days among matched control participants. For the nirmatrelvir-ritonavir group, crude incidence rates of the composite outcome were 695.8 (95% CI, 630.1-766.4) per 100 000 person-days among nirmatrelvir-ritonavir users and 1131.2 (95% CI, 1037.4-1231.2) per 100 000 person-days among matched control participants (Table 2).

**Table 2.** Outcomes for Outpatient Oral Antiviral vs Respective Matched Control Groups

Outcome	Oral antiviral group			Control group			Oral antiviral group vs control group	
	Cumulative incidence of new events, No. (%)	Crude incidence rate		Cumulative incidence of new events, No. (%)	Crude incidence rate		Hazard ratio (95% CI)^a^	*P* value
		Events (95% CI) per 100 000 person-days	Person-days		Events (95% CI) per 100 000 person-days	Person-days		
Molnupiravir (n = 921) vs control (n = 921)								
All-cause mortality and/or hospitalization	592 (64.3)	1024.9 (944.0-1110.8)	57 764	731 (79.4)	1952.4 (1813.4-2099.2)	37 441	0.71 (0.64-0.79)	<.001
All-cause mortality	42 (4.6)	34.5 (24.8-46.6)	121 844	75 (8.1)	70.0 (55.1-87.8)	107 082	0.48 (0.33-0.70)	<.001
All-cause hospitalization	592 (64.3)	1024.9 (944.0-1110.8)	57 764	731 (79.4)	1952.4 (1813.4-2099.2)	37 441	0.71 (0.64-0.79)	<.001
In-hospital disease progression	51 (5.5)	42.8 (31.8-56.2)	119 267	89 (9.7)	85.5 (68.7-105.2)	104 104	0.49 (0.35-0.69)	<.001
Nirmatrelvir-ritonavir (n = 793) vs control (n = 793)								
All-cause mortality and/or hospitalization	411 (51.8)	695.8 (630.1-766.4)	59 072	535 (67.5)	1131.2 (1037.4-1231.2)	47 295	0.71 (0.63-0.80)	<.001
All-cause mortality	8 (1.0)	8.5 (3.7-16.8)	93 678	24 (3.0)	27.5 (17.6-40.9)	87 370	0.29 (0.13-0.63)	.002
All-cause hospitalization	411 (51.8)	695.8 (630.1-766.4)	59 072	535 (67.5)	1131.2 (1037.4-1231.2)	47 295	0.71 (0.63-0.80)	<.001
In-hospital disease progression	39 (4.9)	43.2 (30.7-59.0)	90 370	38 (4.8)	44.1 (31.2-60.6)	86 097	0.92 (0.59-1.44)	.73

^a^ Hazard ratios less than 1 or greater than 1 indicate that oral antiviral users had a higher or lower risk of the outcome compared with the matched control group.

Molnupiravir use was associated with significantly lower risks of composite all-cause mortality and/or hospitalization (HR, 0.71 [95% CI, 0.64-0.79]; *P* < .001), all-cause mortality (HR, 0.48 [95% CI, 0.33-0.70]; *P* < .001), all-cause hospitalization (HR, 0.71 [95% CI, 0.64-0.79]; *P* < .001), and in-hospital disease progression (HR, 0.49 [95% CI, 0.35-0.69]; *P* < .001) compared with nonuse.

Nirmatrelvir-ritonavir use was associated with significantly lower risks of composite all-cause mortality and/or hospitalization (HR, 0.71 [95% CI, 0.63-0.80]; *P* < .001), all-cause mortality (HR, 0.29 [95% CI, 0.13-0.63]; *P* = .002), and all-cause hospitalization (HR, 0.71 [95% CI, 0.63-0.80]; *P* < .001) than nonuse. Furthermore, a nonsignificant lower risk of in-hospital disease progression (HR, 0.92 [95% CI, 0.59-1.44]; *P* = .73) was observed among nirmatrelvir-ritonavir users compared with nonusers.

### Subgroup and Sensitivity Analyses

In subgroup analyses (eTable 2 in Supplement 1), molnupiravir and nirmatrelvir-ritonavir users showed consistently lower risk of composite all-cause mortality and/or hospitalization compared with matched control participants, regardless of age, sex, baseline insulin use, and COVID-19 vaccination status. Although the *P* value for interaction of treatment effect of nirmatrelvir-ritonavir across age groups was less than .05, the treatment effects of nirmatrelvir-ritonavir for younger (≤65 years) and older (>65 years) patients could not be directly compared because the incidence of outcomes in treatment and control groups was lower among younger patients compared with older patients. Nonetheless, it showed a gradation of protection by nirmatrelvir-ritonavir for younger and older patients with differential clinical profiles and disease severity. Results of subgroup analyses of patients with chronic kidney disease and diabetic complications, however, did not reach statistical significance. All sensitivity analyses showed consistent results as in the main analysis (eTable 3 in Supplement 1).

## Discussion

This cohort study examined the effectiveness of molnupiravir and nirmatrelvir-ritonavir exclusively among patients with COVID-19 and comorbid type 2 diabetes in a contemporary cohort during the Omicron wave in Hong Kong. Both oral antiviral agents were associated with a statistically significant 29% reduction in all-cause mortality and hospitalization among patients with type 2 diabetes compared with control participants who did not receive either antiviral medication. Furthermore, we observed that both oral antiviral medications reduced the risk of in-patient disease progression. These findings are notable because diabetes is a common indication for antiviral prescribing for COVID-19 management.

The landmark MOVe-OUT trial reported that molnupiravir use was associated with a reduction in hospitalization or mortality among nonhospitalized unvaccinated patients with COVID-19.^7^ In the MOVe-OUT trial, 228 participants (15.9%) had diabetes (107 in molnupiravir group and 121 in placebo group). In the diabetes subgroup, the absolute risk difference in the incidence of hospitalization or death was 1.4% (95% CI, −8.2 to 11.1), favoring placebo over molnupiravir among patients with diabetes. It is possible that a small subgroup of patients with diabetes contributed to this result in the subgroup analysis. Apart from the landmark trial, 2 additional studies^23,24^ of molnupiravir use included a proportion of patients with diabetes. In a retrospective population-based study showing that molnupiravir use was not associated with a reduction in hospitalization in general, 13.1% of the cohort had diabetes.^23^ The PANORAMIC study was a randomized clinical trial showing that molnupiravir use among high-risk vaccinated individuals with SARS-CoV-2 infection did not reduce the already-low hospitalizations and deaths, but resulted in faster time to recovery and reduced viral detection and load.^24^ In the PANORAMIC trial, 12% of the cohort had diabetes. Subgroup analysis stratified by diabetes status was only performed in the PANORAMIC trial, suggesting no significant interaction by diabetes status (*P* = .12). However, the PANORAMIC cohort was younger (mean age, 56.6 years) and most participants had received 3 doses of COVID-19 vaccine; therefore, the results may not be generalizable to the population most at risk of hospitalization and death from COVID-19 infection.^25^ To our knowledge, our study is the first to specifically evaluate a group of molnupiravir-treated patients with type 2 diabetes, and we observed that molnupiravir use was associated with a lower risk of all-cause mortality and hospitalization and further benefit in in-hospital disease progression. Furthermore, a beneficial effect was observed across a broad spectrum of patients with type 2 diabetes, stratified by age, sex, vaccination status, and baseline insulin use—factors commonly identified as prognostic factors of COVID-19 outcomes.^26^ In considering prescribing of oral antiviral medications, patients with type 2 diabetes often have cardiovascular comorbidities with potential drug-drug interactions between nirmatrelvir-ritonavir and essential cardiovascular medications.^27^ Our findings suggest that in scenarios in which patients with type 2 diabetes are not eligible to receive nirmatrelvir-ritonavir, molnupiravir may be a reasonable option.

The landmark EPIC-HR trial reported that nirmatrelvir-ritonavir use was associated with reduced hospitalization or mortality among nonhospitalized unvaccinated patients with COVID-19.^8^ In the EPIC-HR trial, approximately 10% of participants had type 2 diabetes. In subgroup analysis of efficacy among patients with diabetes, a beneficial effect was observed with nirmatrelvir-ritonavir use compared with placebo, resulting in an estimated absolute risk reduction of −5.51% (95% CI, −10.51 to −0.52). Subsequent retrospective cohort studies provided evidence of the effectiveness of nirmatrelvir-ritonavir. Subgroup analysis concerning diabetes status was performed by Najjar-Debbiny et al,^28^ showing that nirmatrelvir-ritonavir use was associated with a reduced risk of severe COVID-19 or mortality among 1826 patients with diabetes (HR, 0.44 [95% CI, 0.25-0.75]). Our study showed consistent results among 793 nirmatrelvir-ritonavir users with diabetes, demonstrating a 29% reduction in risk of hospitalization and all-cause mortality. The fact that our study cohort was matched for various diabetes-specific characteristics could account for the difference in HR magnitude compared with that obtained by Najjar-Debbiny et al.^28^ Subgroup analyses in our study provided further insight into potential differential benefits in certain groups of patients with type 2 diabetes. In our study, nirmatrelvir-ritonavir use was associated with benefit regardless of age, sex, vaccination status, and baseline insulin use. Nirmatrelvir-ritonavir use was also associated with a lower risk of in-hospital disease progression, although this did not reach statistical significance. This finding was likely attributable to the better health status of the nirmatrelvir-ritonavir cohort, as evidenced by fewer events of in-hospital disease progression.

Of note, for both oral antiviral medications studied here, results for the subgroup with chronic kidney disease did not reach statistical significance, as the sample size was relatively small (3.7% in the molnupiravir cohort and 0.3% in the nirmatrelvir-ritonavir cohort). This could be explained by concern regarding kidney function owing to nirmatrelvir-ritonavir use. Similarly, for both oral antiviral agents, HRs in the subgroup with diabetic complications did not reach statistical significance. In fact, the presence of diabetic complications, especially microvascular complications, has been established as a risk factor for severe COVID-19.^29^ It has been postulated that underlying systemic endothelial and microvascular dysfunction may lead to excessive pulmonary endothelial and microvascular injury upon SARS-CoV-2 infection and thus to worse COVID-19 outcomes.^29^ Whether this may explain the attenuation of effectiveness of oral antiviral medications warrants further evaluation. The sample size of this subgroup was also relatively small. Hence, our results encourage future studies with larger sample sizes to clarify the effectiveness of oral antiviral agents among patients with type 2 diabetes and concomitant chronic kidney disease or diabetic complications.

### Limitations

Our cohort study included a nonselective population with confirmed SARS-CoV-2 infection in the local community consisting exclusively of patients with type 2 diabetes amid the Omicron wave in Hong Kong, and we observed clinical effectiveness of both molnupiravir and nirmatrelvir-ritonavir oral antiviral medications in the contemporary setting. Nonetheless, our results should be interpreted bearing certain limitations. First, we could not entirely exclude the possibility that patients had COVID-19 but were unaware of their infection status because they had not tested, their rapid antigen testing had suboptimal sensitivity, or they had not reported their infection status to the local health authorities. Hence, these patients would not be eligible for oral antiviral treatment. Second, we did not further differentiate all-cause mortality into mortality associated with COVID-19 or other causes. Third, laboratory parameters, such as glycated hemoglobin A_1c_ and eGFR, were only available for a small proportion of patients in this data set. Nonetheless, we matched participants according to a multitude of diabetes-specific variables, including diabetic complications and antidiabetic regimes, to ensure balance in baseline characteristics between antiviral-treated patients and control participants in terms of glycemic control and diabetes severity. Fourth, we limited the scope of the study population to community-dwelling patients with type 2 diabetes who were not institutionalized, which might limit the generalizability of our results. Fifth, although all hospitals under the Hospital Authority in Hong Kong shared the same standard care protocol for COVID-19, including hospital admission criteria, there may be variations in hospital admission criteria in other parts of the world such that our findings may not entirely apply. Nonetheless, we observed that oral antiviral medications were associated with reduced mortality, a hard clinical outcome. Sixth, the primary cause of hospital admission and information regarding drug consumption could not be ascertained from the electronic health database used in our study. Finally, despite attempts to control for a range of baseline covariates, the usual caveats of residual and unmeasured confounding about observational studies apply.

## Conclusions

The findings of this cohort study suggest that oral antiviral use was associated with a 29% lower risk of all-cause mortality and/or hospitalization for both molnupiravir users and nirmatrelvir-ritonavir users with type 2 diabetes and SARS-CoV-2 infection during the Omicron wave in Hong Kong. These findings support the effectiveness of either oral antiviral drug in the outpatient setting. Further studies in specific populations, such as individuals in residential care homes and individuals with chronic kidney disease, are suggested.

## Notes

Supplement 1. **eTable 1.** Baseline Characteristics of Patients With Type 2 Diabetes and COVID-19 in Molnupiravir, Nirmatrelvir-Ritonavir, and Control Groups Before 1:1 Propensity-Score Matching **eTable 2.** Subgroup Analyses of the Primary Outcome for Outpatient Oral Antiviral vs Respective Matched Control Groups **eTable 3.** Sensitivity Analyses of the Primary Outcome **eTable 4.** Definition of Covariates **eFigure 1.** Distribution of Propensity Scores in Oral Antiviral and Respective Matched Control Groups Before and After Propensity Score Matching **eFigure 2.** Cumulative Incidence Plots of In-Hospital Disease Progression for (A) Outpatient Molnupiravir Users vs Matched Controls and (B) Outpatient Nirmatrelvir-Ritonavir Users vs Matched Controls

Supplement 2.
Data Sharing Statement
