# Supplementary material for: Analysis of All-Cause Hospitalization and Death Among Nonhospitalized Patients With Type 2 Diabetes and SARS-CoV-2 Infection Treated With Molnupiravir or Nirmatrelvir-Ritonavir During the Omicron Wave in Hong Kong
Source: JAMA Netw Open. 2023 May 19;6(5):e2314393. doi: 10.1001/jamanetworkopen.2023.14393 (PMC10199353; doi:10.1001/jamanetworkopen.2023.14393)
Supplement: Supplement 1. — eTable 1. Baseline Characteristics of Patients With Type 2 Diabetes and COVID-19 in Molnupiravir, Nirmatrelvir-Ritonavir, and Control Groups Before 1:1 Propensity-Score Matching eTable 2. Subgroup Analyses of the Primary Outcome for Outpatient Oral Antiviral vs Respective Matched Control Groups eTable 3. Sensitivity Analyses of the Primary Outcome eTable 4. Definition of Covariates eFigure 1. Distribution of Propensity Scores in Oral Antiviral and Respective Matched Control Groups Before and After Propensity Score Matching eFigure 2. Cumulative Incidence Plots of In-Hospital Disease Progression for (A) Outpatient Molnupiravir Users vs Matched Controls and (B) Outpatient Nirmatrelvir-Ritonavir Users vs Matched Controls [file jamanetwopen-e2314393-s001.pdf]

Supplementary Online Content

Lui DTW, Chung MSH, Lau EHY, et al. Analysis of all-cause hospitalization and death among nonhospitalized patients with type 2 diabetes and SARS-CoV-2 infection treated with molnupiravir or nirmatrelvir-ritonavir during the Omicron wave in Hong Kong. *JAMA Netw Open*. 2023;6(5):e2314393. doi:10.1001/jamanetworkopen.2023.14393

eTable 1. Baseline Characteristics of Patients With Type 2 Diabetes and COVID-19 in Molnupiravir, Nirmatrelvir-Ritonavir, and Control Groups Before 1:1 Propensity Score Matching

eTable 2. Subgroup Analyses of the Primary Outcome for Outpatient Oral Antiviral vs Respective Matched Control Groups

eTable 3. Sensitivity Analyses of the Primary Outcome

eTable 4. Definition of Covariates

eFigure 1. Distribution of Propensity Score in Oral Antiviral and Respective Matched Control Groups Before and After Propensity Score Matching

eFigure 2. Cumulative Incidence Plots of In-Hospital Disease Progression for (A) Outpatient Molnupiravir Users vs Matched Controls and (B) Outpatient Nirmatrelvir-Ritonavir Users vs Matched Controls

This supplementary material has been provided by the authors to give readers additional information about their work.

© 2023 Lui DTW et al. *JAMA Network Open*.

eTable 1. Baseline Characteristics of Patients With Type 2 Diabetes and COVID-19 in Molnupiravir, Nirmatrelvir-Ritonavir, and Control Groups Before 1:1 Propensity Score Matching

Baseline characteristics	Molnupiravir (N=950)		Nirmatrelvir/ ritonavir (N=994)		Control (N=5,539)		SMD (Molnupiravir vs Control)	SMD (Nirmatrelvir/ ritonavir vs Control)
	N / Mean	% / SD	N / Mean	% / SD	N / Mean	% / SD		
Age, years *	76.9	10.8	72.1	11.1	72.5	12.8	0.36	0.03
18-40	6	(0.6%)	6	(0.6%)	83	(1.5%)		
41-65	118	(12.4%)	243	(24.5%)	1,490	(26.9%)	0.39	0.11
>65	826	(87.0%)	745	(75.0%)	3,966	(71.6%)		
Sex							0.05	0.13
Male	498	(52.4%)	479	(48.2%)	3,034	(54.8%)		
Female	452	(47.6%)	515	(51.8%)	2,505	(45.2%)		
Time of SARS-CoV-2 infection								
Before 1st September 2022	240	(25.3%)	368	(37.0%)	338	(6.1%)	0.55	0.81
On or after 1st September 2022	710	(74.7%)	626	(63.0%)	5,201	(93.9%)		
Pre-existing comorbidities								
Charlson Comorbidity Index * †	5.7	1.6	4.9	1.3	5.5	1.9	0.12	0.32
0-4	153	(16.1%)	350	(35.2%)	1,549	(28.0%)		
5-6	557	(58.6%)	554	(55.7%)	2,584	(46.7%)	0.31	0.44
7-16	240	(25.3%)	90	(9.1%)	1,406	(25.4%)		
Hypertension	879	(92.5%)	824	(82.9%)	4,792	(86.5%)	0.20	0.10
Chronic lung disease	120	(12.6%)	61	(6.1%)	913	(16.5%)	0.11	0.33
Chronic heart disease	247	(26.0%)	132	(13.3%)	1,270	(22.9%)	0.07	0.25
Chronic kidney disease	56	(5.9%)	9	(0.9%)	362	(6.5%)	0.03	0.30
Liver disease	37	(3.9%)	36	(3.6%)	284	(5.1%)	0.06	0.07
Malignancy	46	(4.8%)	33	(3.3%)	386	(7.0%)	0.09	0.17
Gout	21	(2.2%)	12	(1.2%)	177	(3.2%)	0.06	0.14
Obesity	61	(6.4%)	67	(6.7%)	509	(9.2%)	0.10	0.09

© 2023 Lui DTW et al. *JAMA Network Open*.

Obstructive sleep apnea	0	(0.0%)	0	(0.0%)	0	(0.0%)	NA	NA
Depression	1	(0.1%)	0	(0.0%)	23	(0.4%)	0.06	0.09
Severe hypoglycemia	73	(7.7%)	49	(4.9%)	565	(10.2%)	0.09	0.20
Diabetic complications	282	(29.7%)	142	(14.3%)	1,608	(29.0%)	0.01	0.36
Macrovascular diseases	266	(28.0%)	132	(13.3%)	1,509	(27.2%)	0.02	0.35
Coronary heart disease	133	(14.0%)	67	(6.7%)	727	(13.1%)	0.03	0.21
Heart failure	68	(7.2%)	12	(1.2%)	420	(7.6%)	0.02	0.31
Stroke	115	(12.1%)	64	(6.4%)	631	(11.4%)	0.02	0.17
Peripheral vascular disease	6	(0.6%)	4	(0.4%)	50	(0.9%)	0.03	0.06
Microvascular diseases	29	(3.1%)	13	(1.3%)	185	(3.3%)	0.02	0.14
Non-proliferative diabetic retinopathy	22	(2.3%)	9	(0.9%)	125	(2.3%)	0.00	0.11
Sight threatening diabetic retinopathy	9	(0.9%)	4	(0.4%)	65	(1.2%)	0.02	0.09
Neuropathy	4	(0.4%)	3	(0.3%)	41	(0.7%)	0.04	0.06
End-stage renal disease	0	(0.0%)	0	(0.0%)	0	(0.0%)	NA	NA
SARS-CoV-2 vaccination status ‡								
Not fully vaccinated	545	(57.4%)	363	(36.5%)	3,999	(72.2%)	0.33	0.79
Fully vaccinated but not boosted	262	(27.6%)	386	(38.8%)	1,139	(20.6%)		
Boosted	143	(15.1%)	245	(24.7%)	401	(7.2%)		
Number of doses received								
0-2	339	(35.7%)	271	(27.3%)	2,913	(52.6%)	0.35	0.54
≥3	611	(64.3%)	723	(72.7%)	2,626	(47.4%)		
Long-term medications								
ACEI/ARB	589	(62.0%)	497	(50.0%)	3,141	(56.7%)	0.11	0.13
Anticoagulants	255	(26.8%)	92	(9.3%)	1,542	(27.8%)	0.02	0.49
Antiplatelets	379	(39.9%)	256	(25.8%)	1,958	(35.3%)	0.09	0.21
Lipid-lowering agents	704	(74.1%)	637	(64.1%)	3,747	(67.6%)	0.14	0.08
NSAID	451	(47.5%)	351	(35.3%)	2,561	(46.2%)	0.02	0.22
Beta-blockers	382	(40.2%)	254	(25.6%)	2,054	(37.1%)	0.06	0.25
Calcium-channel blockers	622	(65.5%)	527	(53.0%)	3,329	(60.1%)	0.11	0.14
Diuretics	290	(30.5%)	152	(15.3%)	1,720	(31.1%)	0.01	0.38
Anti-depressants	104	(10.9%)	40	(4.0%)	431	(7.8%)	0.11	0.16

© 2023 Lui DTW et al. *JAMA Network Open*.

Antidiabetic regimen								
Non-insulin users								
Single agent	219	(23.1%)	284	(28.6%)	1,080	(19.5%)		
2 agents	138	(14.5%)	169	(17.0%)	631	(11.4%)	0.18	0.43
>2 agents	85	(9.0%)	92	(9.3%)	395	(7.1%)		
Insulin	355	(37.4%)	226	(22.7%)	2,322	(41.9%)	0.09	0.42
Antidiabetic medications								
GLP1RA	14	(1.5%)	12	(1.2%)	53	(1.0%)	0.05	0.02
Metformin	637	(67.1%)	728	(73.2%)	3,703	(66.9%)	0.00	0.14
SU	422	(44.4%)	356	(35.8%)	2,364	(42.7%)	0.04	0.14
TZD	75	(7.9%)	91	(9.2%)	545	(9.8%)	0.07	0.02
Acarbose	5	(0.5%)	6	(0.6%)	22	(0.4%)	0.02	0.03
SGLT2i	115	(12.1%)	69	(6.9%)	516	(9.3%)	0.09	0.09
DPP4i	265	(27.9%)	175	(17.6%)	1,348	(24.3%)	0.08	0.17

Notes: ACEI/ARB = angiotensin-converting enzyme inhibitors / angiotensin receptor blockers; DPP4i = dipeptidyl peptidase 4 inhibitors; GLP1RA = glucagon-like peptide 1 receptor agonists; NA = not applicable; NSAID = non-steroidal anti-inflammatory drugs; SD = standard deviation; SGLT2i = sodium-glucose cotransporter-2 inhibitors; SMD = standardized mean difference; SU = sulfonylureas; TZD = thiazolidinediones

* Age, and Charlson Comorbidity index are presented in mean ± SD

† The calculation of Charlson Comorbidity Index does not include Acquired Immune Deficiency Syndrome

‡ Fully vaccinated but not boosted patients were defined as those with 2 doses of BNT162b2 (Comirnaty) or 3 doses of COVID-19

© 2023 Lui DTW et al. *JAMA Network Open*.

Vaccine (Vero Cell), Inactivated (CoronaVac); boosted patients were defined as those with at least 3 doses of BNT162b2 (Comirnaty) or 4 doses of COVID-19 Vaccine (Vero Cell), Inactivated. (CoronaVac).

© 2023 Lui DTW et al. *JAMA Network Open*.

eTable 2. Subgroup Analyses of the Primary Outcome for Outpatient Oral Antiviral vs Respective Matched Control Groups

Outcomes	Oral antiviral						Control						Oral antiviral vs Control			
	Cumulative incidence			Crude incidence rate (Events / 100,000 person-days)			Cumulative incidence			Crude incidence rate (Events / 100,000 person-days)						
	N	New events	Rate	Estimate	95% CI	Person-days	N	New events	Rate	Estimate	95% CI	Person-days	HR§	95% CI	P-value	P_{int}
**Molnupiravir vs Control**																
All-cause mortality or hospitalization	921	592	64.3%	1,024.9	(944.0, 1,110.8)	57,764	921	731	79.4%	1,952.4	(1,813.4, 2,099.2)	37,441	0.71	(0.64, 0.79)	<0.001	
Age ≤65 years	115	58	50.4%	641.2	(486.9, 828.9)	9,046	115	84	73.0%	1,398.1	(1,115.2, 1,731.0)	6,008	0.62	(0.45, 0.86)	0.005	0.317
Age >65 years	806	538	66.7%	1,109.6	(1,017.8, 1,207.5)	48,484	806	638	79.2%	1,850.3	(1,709.5, 1,999.6)	34,481	0.75	(0.68, 0.84)	<0.001	
Male	466	318	68.2%	1,240.4	(1,107.8, 1,384.6)	25,636	466	384	82.4%	2,390.6	(2,157.4, 2,642.1)	16,063	0.72	(0.62, 0.83)	<0.001	0.508
Female	427	255	59.7%	808.3	(712.1, 913.8)	31,548	427	324	75.9%	1,558.6	(1,393.5, 1,737.9)	20,788	0.67	(0.58, 0.79)	<0.001	
Fully vaccinated	361	237	65.7%	1,602.3	(1,404.8, 1,819.8)	14,791	361	297	82.3%	3,607.9	(3,209.2, 4,042.4)	8,232	0.69	(0.58, 0.81)	<0.001	0.504
Not-fully vaccinated/ unvaccinated	533	335	62.9%	786.9	(704.9, 875.8)	42,573	533	406	76.2%	1,286.9	(1,164.7, 1,418.4)	31,549	0.74	(0.65, 0.86)	<0.001	
Insulin users	339	298	87.9%	3,452.3	(3,071.4, 3,867.3)	8,632	339	327	96.5%	8,932.0	(7,990.0, 9,954.5)	3,661	0.74	(0.64, 0.85)	<0.001	0.069
Non-insulin users	559	280	50.1%	577.3	(511.7, 649.0)	48,501	559	385	68.9%	1,128.8	(1,018.8, 1,247.4)	34,107	0.65	(0.56, 0.75)	<0.001	
With diabetic complications	254	221	87.0%	2,829.7	(2,468.9, 3,228.4)	7,810	254	225	88.6%	2,810.7	(2,455.5, 3,203.0)	8,005	1.00	(0.84, 1.18)	0.960	<0.001
Without diabetic complications	638	352	55.2%	712.9	(640.4, 791.4)	49,373	638	460	72.1%	1,368.6	(1,246.4, 1,499.6)	33,611	0.67	(0.59, 0.76)	<0.001	
Presence of chronic kidney disease	34	34	100.0%	11,409.4	(7,901.3, 15,943.5)	298	34	34	100.0%	13,821.1	(9,571.5, 19,313.7)	246	0.81	(0.48, 1.34)	0.407	0.432
Without presence of chronic kidney disease	870	541	62.2%	942.5	(864.8, 1,025.4)	57,398	870	652	74.9%	1,506.1	(1,392.7, 1,626.2)	43,292	0.76	(0.68, 0.84)	<0.001	
**Nirmatrelvir/ritonavir vs Control**																
All-cause mortality or hospitalization	793	411	51.8%	695.8	(630.1, 766.4)	59,072	793	535	67.5%	1,131.2	(1,037.4, 1,231.2)	47,295	0.71	(0.63, 0.80)	<0.001	
Age ≤65 years	207	63	30.4%	315.7	(242.6, 404.0)	19,953	207	119	57.5%	758.8	(628.6, 908.1)	15,682	0.46	(0.34, 0.62)	<0.001	0.006
Age >65 years	568	329	57.9%	854.5	(764.6, 952.0)	38,504	568	400	70.4%	1,271.6	(1,150.0, 1,402.6)	31,456	0.76	(0.67, 0.86)	<0.001	
Male	380	187	49.2%	623.3	(537.2, 719.4)	30,000	380	270	71.1%	1,301.8	(1,151.1, 1,466.7)	20,741	0.61	(0.51, 0.72)	<0.001	0.314
Female	396	200	50.5%	694.5	(601.6, 797.7)	28,797	396	261	65.9%	1,083.0	(955.6, 1,222.7)	24,100	0.70	(0.59, 0.83)	<0.001	
Fully vaccinated	458	240	52.4%	1,010.6	(886.8, 1,146.9)	23,748	458	338	73.8%	2,035.0	(1,823.8, 2,264.0)	16,609	0.62	(0.52, 0.73)	<0.001	0.740
Not-fully vaccinated/ unvaccinated	321	143	44.5%	403.1	(339.7, 474.8)	35,476	321	204	63.6%	778.6	(675.4, 893.1)	26,201	0.61	(0.51, 0.72)	<0.001	
Insulin users	202	162	80.2%	2,458.3	(2,094.3, 2,867.3)	6,590	202	189	93.6%	6,774.2	(5,842.8, 7,811.8)	2,790	0.75	(0.61, 0.91)	0.004	0.031

© 2023 Lui DTW et al. *JAMA Network Open*.

Non-insulin users	579	234	40.4%	447.8	(392.3, 509.1)	52,250	579	356	61.5%	886.9	(797.2, 984.0)	40,138	0.58	(0.50, 0.68)	<0.001	
With diabetic complications	112	88	78.6%	1,918.9	(1,539.0, 2,364.1)	4,586	112	100	89.3%	3,012.0	(2,450.7, 3,663.5)	3,320	0.81	(0.61, 1.06)	0.119	0.144
Without diabetic complications	659	293	44.5%	539.3	(479.3, 604.8)	54,326	659	410	62.2%	903.7	(818.3, 995.6)	45,369	0.65	(0.57, 0.75)	<0.001	
Presence of chronic kidney disease	2	2	100.0%	20,000.0	(2,422.1, 72,246.9)	10	2	2	100.0%	14,285.7	(1,730.1, 51,604.9)	14	NA	NA	NA	<0.001
Without presence of chronic kidney disease	785	397	50.6%	673.3	(608.7, 742.9)	58,963	785	549	69.9%	1,251.2	(1,148.7, 1,360.3)	43,879	0.64	(0.57, 0.72)	<0.001	

Notes: CI = confidence interval; HR = hazard ratio; NA = not applicable; P_{int} = P-value for interaction

§ HR >1 (or <1) indicates oral antiviral users had higher (lower) risk of outcome compared to the matched control group.

© 2023 Lui DTW et al. *JAMA Network Open*.

eTable 3. Sensitivity Analyses of the Primary Outcome

Outcomes	Oral antiviral					Control					Oral antiviral vs Control		
	Cumulative incidence		Crude incidence rate (Events / 100,000 person-days)			Cumulative incidence		Crude incidence rate (Events / 100,000 person-days)					
	New events	Rate	Estimate	95% CI	Person-days	New events	Rate	Estimate	95% CI	Person-days	HR§	95% CI	P-value
**Molnupiravir vs Control**													
**Molnupiravir users who initiated molnupiravir within 2 days since index date (N=755)**													
All-cause mortality or hospitalization	518	68.6%	1,277.4	(1,169.8, 1,392.3)	40,551	616	81.6%	2,355.4	(2,173.0, 2,548.9)	26,153	0.76	(0.68, 0.84)	<0.001
All-cause mortality	35	4.6%	36.8	(25.6, 51.2)	95,143	58	7.7%	66.6	(50.6, 86.1)	87,046	0.52	(0.34, 0.79)	0.002
Hospitalization	518	68.6%	1,277.4	(1,169.8, 1,392.3)	40,551	616	81.6%	2,355.4	(2,173.0, 2,548.9)	26,153	0.76	(0.68, 0.84)	<0.001
**Limiting the follow-up period to be at most 30 days (N=921)**													
All-cause mortality or hospitalization	577	62.6%	4,087.0	(3,760.3, 4,434.5)	14,118	715	77.6%	6,183.5	(5,738.5, 6,653.9)	11,563	0.71	(0.64, 0.79)	<0.001
All-cause mortality	30	3.3%	113.4	(76.5, 162.0)	26,444	66	7.2%	289.8	(224.1, 368.7)	22,774	0.39	(0.25, 0.60)	<0.001
Hospitalization	577	62.6%	4,087.0	(3,760.3, 4,434.5)	14,118	715	77.6%	6,183.5	(5,738.5, 6,653.9)	11,563	0.71	(0.64, 0.79)	<0.001
**Adjusting propensity score model (excluding variables of pre-comorbidities and use of medications) (N=927)**													
All-cause mortality or hospitalization	599	64.6%	1,035.3	(954.1, 1,121.6)	57,857	754	81.3%	2,151.8	(2,000.9, 2,311.0)	35,041	0.68	(0.61, 0.75)	<0.001
All-cause mortality	44	4.7%	36.0	(26.2, 48.3)	122,230	88	9.5%	83.0	(66.6, 102.3)	105,996	0.43	(0.30, 0.61)	<0.001
Hospitalization	599	64.6%	1,035.3	(954.1, 1,121.6)	57,857	754	81.3%	2,151.8	(2,000.9, 2,311.0)	35,041	0.68	(0.61, 0.75)	<0.001
**Adjusting propensity score model (with a wider caliper width of 0.1 and an increase ratio of 1:2) (N=792)**													
All-cause mortality or hospitalization	494	62.4%	893.6	(816.5, 976.0)	55,283	1,240	78.6%	1,711.7	(1,617.7, 1,809.7)	72,443	0.68	(0.62, 0.75)	<0.001
All-cause mortality	40	5.1%	34.8	(24.8, 47.3)	115,082	140	8.9%	68.1	(57.3, 80.3)	205,711	0.50	(0.35, 0.70)	<0.001
Hospitalization	494	62.4%	893.6	(816.5, 976.0)	55,283	1,240	78.6%	1,711.7	(1,617.7, 1,809.7)	72,443	0.68	(0.62, 0.75)	<0.001
**Including patients who had on drugs contraindicated to nirmatrelvir/ritonavir (N=944)**													
All-cause mortality or hospitalization	615	65.1%	1,060.7	(978.5, 1,147.9)	57,981	737	78.1%	1,800.0	(1,672.4, 1,934.8)	40,945	0.75	(0.68, 0.83)	<0.001
All-cause mortality	47	5.0%	37.9	(27.9, 50.5)	123,862	70	7.4%	63.3	(49.4, 80.0)	110,523	0.57	(0.40, 0.82)	0.003
Hospitalization	615	65.1%	1,060.7	(978.5, 1,147.9)	57,981	737	78.1%	1,800.0	(1,672.4, 1,934.8)	40,945	0.75	(0.68, 0.83)	<0.001

© 2023 Lui DTW et al. *JAMA Network Open*.

**Nirmatrelvir/ritonavir vs Control**													
**Nirmatrelvir/ritonavir users who initiated nirmatrelvir/ritonavir within 2 days since index date (N=677)**													
All-cause mortality or hospitalization	363	53.6%	792.4	(712.9, 878.2)	45,812	473	69.9%	1,308.1	(1,192.9, 1,431.5)	36,159	0.72	(0.63, 0.81)	<0.001
All-cause mortality	8	1.2%	10.5	(4.5, 20.7)	76,291	17	2.5%	23.5	(13.7, 37.6)	72,359	0.41	(0.18, 0.96)	0.040
Hospitalization	363	53.6%	792.4	(712.9, 878.2)	45,812	473	69.9%	1,308.1	(1,192.9, 1,431.5)	36,159	0.72	(0.63, 0.81)	<0.001
**Limiting the follow-up period to be at most 30 days (N=793)**													
All-cause mortality or hospitalization	405	51.1%	2,801.0	(2,534.8, 3,087.6)	14,459	521	65.7%	4,110.1	(3,764.7, 4,478.7)	12,676	0.72	(0.64, 0.81)	<0.001
All-cause mortality	6	0.8%	25.9	(9.5, 56.4)	23,166	18	2.3%	89.0	(52.7, 140.6)	20,226	0.29	(0.11, 0.73)	0.008
Hospitalization	405	51.1%	2,801.0	(2,534.8, 3,087.6)	14,459	521	65.7%	4,110.1	(3,764.7, 4,478.7)	12,676	0.72	(0.64, 0.81)	<0.001
**Adjusting propensity score model (excluding variables of pre-comorbidities and use of medications) (N=824)**													
All-cause mortality or hospitalization	418	50.7%	691.8	(627.0, 761.4)	60,424	584	70.9%	1,334.7	(1,228.6, 1,447.4)	43,756	0.63	(0.56, 0.70)	<0.001
All-cause mortality	8	1.0%	8.4	(3.6, 16.5)	95,313	28	3.4%	32.0	(21.3, 46.3)	87,485	0.24	(0.11, 0.54)	<0.001
Hospitalization	418	50.7%	691.8	(627.0, 761.4)	60,424	584	70.9%	1,334.7	(1,228.6, 1,447.4)	43,756	0.63	(0.56, 0.70)	<0.001
**Adjusting propensity score model (with a wider caliper width of 0.1 and an increase ratio of 1:2) (N=639)**													
All-cause mortality or hospitalization	324	50.7%	605.6	(541.4, 675.2)	53,503	852	67.2%	1,005.1	(938.7, 1,074.9)	84,770	0.68	(0.61, 0.77)	<0.001
All-cause mortality	8	1.3%	9.5	(4.1, 18.8)	83,892	44	3.5%	27.6	(20.0, 37.0)	159,621	0.32	(0.16, 0.65)	0.002
Hospitalization	324	50.7%	605.6	(541.4, 675.2)	53,503	852	67.2%	1,005.1	(938.7, 1,074.9)	84,770	0.68	(0.61, 0.77)	<0.001

Notes: CI = confidence interval; HR = hazard ratio

§ HR >1 (or <1) indicates oral antiviral users had higher (lower) risk of outcome compared to the matched control group.

© 2023 Lui DTW et al. *JAMA Network Open*.

eTable 4. Definition of Covariates

	ICD-9-CM codes	ICPC-9 codes	Drug treatment: BNF codes	Drug treatment: searching terms	Clinical parameter
Pre-existing comorbidities					
Hypertension	401-405	K86-K87	2.5.5.1; 2.5.5.2 (ACEI/ARB); 2.4; 2.6.2; 2.2.1; 2.2.2		
Chronic lung disease	490-496	R79 R95		"SALBUTAMOL (SULPHATE)"; "MONTELUKAST (SODIUM)"; "SALBUTAMOL"; "MONTELUKAST"	
Chronic heart disease	410-414	K74-K76		"GLYCERYL TRINITRATE"; "CLOPIDOGREL (HYDROGEN SULPHATE)"; "IMDUR"; "ISOSORBIDE MONONITRATE"; "CLOPIDOGREL"	
Chronic kidney disease	585-586				
Liver disease	155.x; 570-573.9 (only include those who had record before index date)	D97 (only include those who had record before index date)		"ENTECAVIR"; "URSODEOXYCHOLIC ACID [250MG/12.5ML]" (only include those who had record before index date)	
Malignancy	140-209	A79; B72-B74; D74-D77; F74; H75; K72; L71; N74; R84-R85; S77; T71, T73; U75-U77; W72; X75-X77; Y78			
Gout	274.x				
Obesity	278.0x	T82			
Obstructive sleep apnea	327.23				
Depression	311.x				
Severe hypoglycemia	251.0-251.2; 250.8.x				

© 2023 Lui DTW et al. *JAMA Network Open*.

Diabetic complications			
Macrovascular diseases			
Coronary heart disease	410-414	K74-K76	
Heart failure	428.x	K77	
Stroke	430-438.9	K89-K91	
Peripheral vascular disease	250.6x; 440.2x; 997.2; 997.6	K92	
Microvascular diseases			
Non-proliferative diabetic retinopathy	362.01, 362.03-362.06	F83	
Sight threatening diabetic retinopathy	249.5x; 362.02, 362.07		
Neuropathy	249.6x; 250.6x; 337.1x; 355.x; 357.2	N94	
End-stage renal disease			eGFR < 15mL/min/1.73m²

© 2023 Lui DTW et al. *JAMA Network Open*.

eFigure 1. Distribution of Propensity Scores in Oral Antiviral and Respective Matched Control Groups Before and After Propensity Score Matching

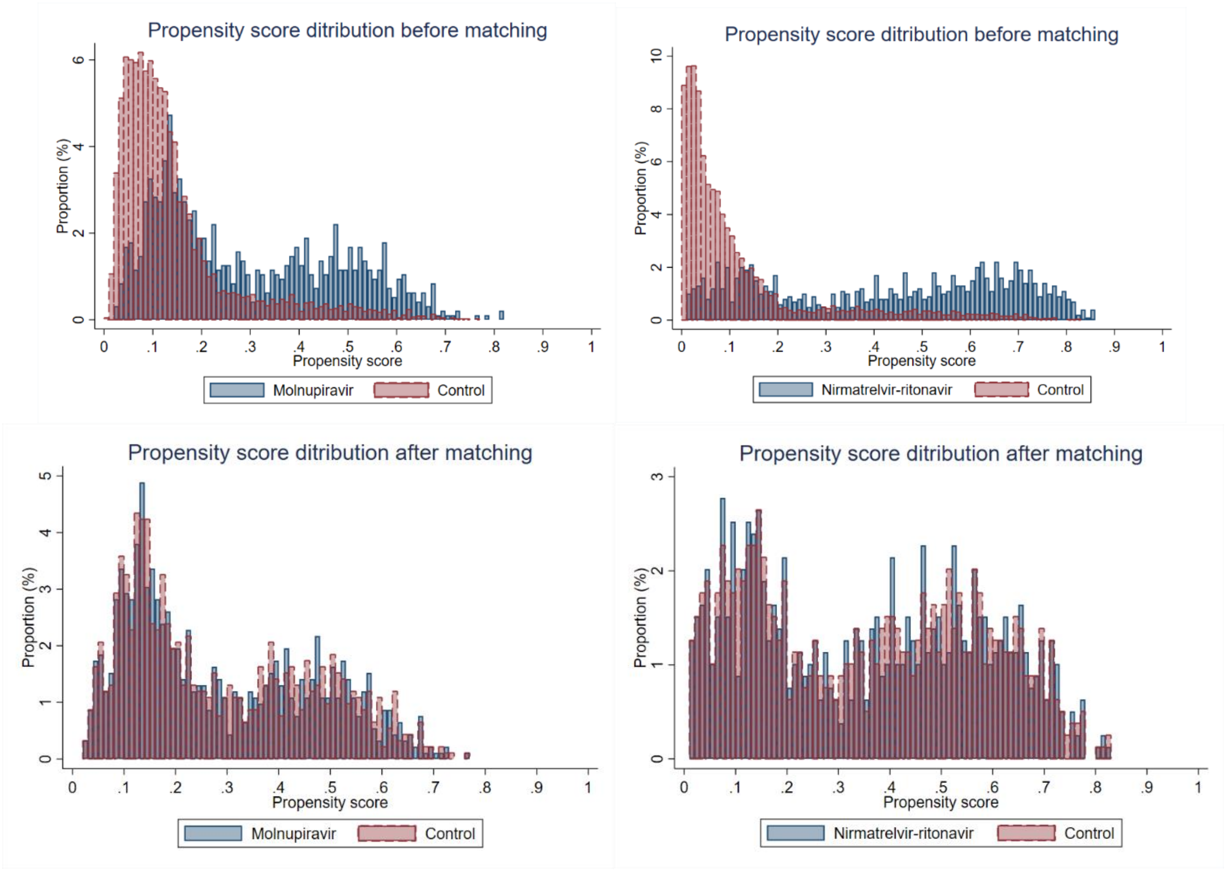

© 2023 Lui DTW et al. *JAMA Network Open*.

eFigure 2. Cumulative Incidence Plots of In-Hospital Disease Progression for (A) Outpatient Molnupiravir Users vs Matched Controls and (B) Outpatient Nirmatrelvir-Ritonavir Users vs Matched Controls

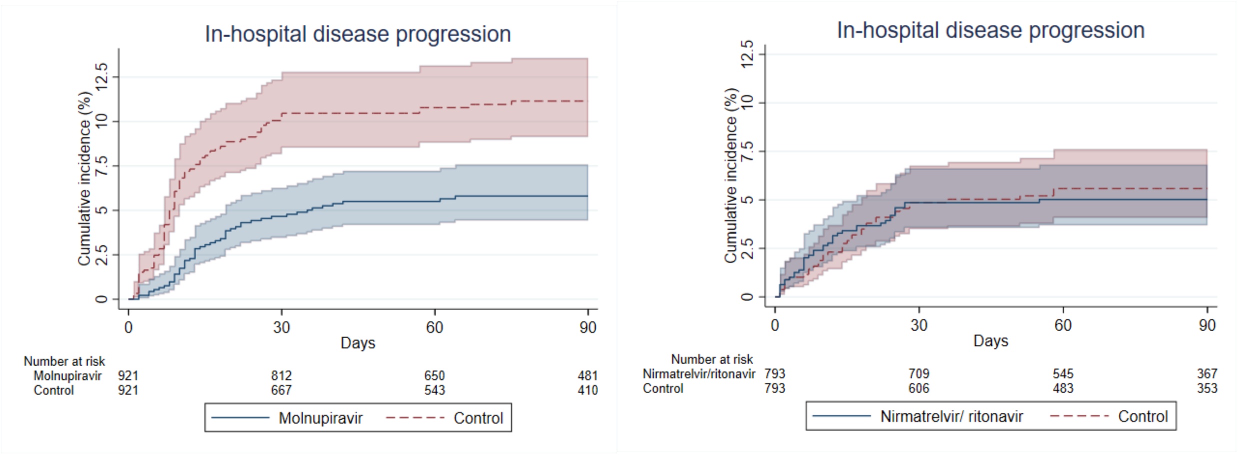

© 2023 Lui DTW et al. *JAMA Network Open*.
