# Supplementary material for: Analysis of All-Cause Hospitalization and Death Among Nonhospitalized Patients With Type 2 Diabetes and SARS-CoV-2 Infection Treated With Molnupiravir or Nirmatrelvir-Ritonavir During the Omicron Wave in Hong Kong
Source: JAMA Netw Open. 2023 May 19;6(5):e2314393. doi: 10.1001/jamanetworkopen.2023.14393 (PMC10199353; doi:10.1001/jamanetworkopen.2023.14393)
Supplement: Supplement 2. — Data Sharing Statement [file jamanetwopen-e2314393-s002.pdf]

Data Sharing Statement

Lui. Analysis of All-Cause Hospitalization and Death Among Nonhospitalized Patients With Type 2 Diabetes and SARS-CoV-2 Infection Treated With Molnupiravir or Nirmatrelvir-Ritonavir During the Omicron Wave in Hong Kong. *JAMA Netw Open*. Published May 19, 2023. doi:10.1001/jamanetworkopen.2023.14393

Data
Data available: No

Explanation for why data are not available: The clinical outcome data and vaccination records were extracted from the Hospital Authority database in Hong Kong and data on confirmed cases of SARS-CoV-2 infection were extracted from the eSARS data provided by the Centre for Health Protection (Department of Health, The Government of the Hong Kong Special Administrative Region). The data custodians (the Hospital Authority and the Department of Health) provided the underlying individual patient data to The University of Hong Kong for the purpose of performing scientific research for the study. Restrictions apply to the availability of these data, which were used under license of the Hospital Authority and the Department of Health for this study. The authors cannot transmit or release the data, in whole or in part in whatever form or media, or to any other parties or place outside Hong Kong; and the authors fully comply with the duties under the laws of Hong Kong relating to the protection of personal data including those under the Personal Data (Privacy) Ordinance and its principles in all aspects.
